# Analysis of experiences with exclusive breastfeeding among HIV-positive mothers in Lusaka, Zambia

**DOI:** 10.3402/gha.v9.32362

**Published:** 2016-12-01

**Authors:** Alice Ngoma-Hazemba, Busisiwe Purity Ncama

**Affiliations:** 1 Department of Public Health, School of Medicine, University of Zambia, Lusaka, Zambia; 2 School of Nursing and Public Health, University of KwaZulu-Natal, Durban, South Africa

**Keywords:** counselling, infant feeding, mother-to-child transmission, behaviour, breastfeeding, experience, HIV-free survival, wellbeing

## Abstract

**Background:**

Exclusive breastfeeding in the first 6 months offers protection from postnatal HIV infection but remains low in resource-poor settings. Documentation of experiences with exclusive breastfeeding is needed to strengthen infant feeding counselling by health care workers.

**Objective:**

To explore HIV-positive mothers’ experiences with exclusive breastfeeding during the first 6 months of the infant's life.

**Design:**

A health facility- and community-based exploratory qualitative study was conducted among 30 HIV-positive mothers practising exclusive breastfeeding and meeting the selection criteria. In-depth interviews were conducted four times with the same participants at 6 days, 6 weeks, 12 weeks and 18 weeks after giving birth. Interviews were transcribed verbatim and files were imported into QRS NVivo Version 10 for coding, and data were analysed using the framework analysis.

**Results:**

We found that mothers were aware of the risks of mother-to-child transmission of HIV through breastmilk and recognised the benefits of exclusive breastfeeding to their exposed babies. However, they were particularly concerned about achieving HIV-free survival for their exposed infants because of problems faced during the first 6 months of infant feeding. Although they reported being supported by their families and/or friends, their poor health and nutrition impacted how well they cared for their infants’ well-being.

**Conclusions:**

We conclude that exclusive breastfeeding was difficult to achieve because of individual circumstances. Therefore, prevention of mother-to-child transmission interventions that adopt a once-off infant feeding counselling do not achieve adequate preparation on the part of mothers to practice exclusive breastfeeding. There is a need to provide frontline health care workers with steps for consideration during infant feeding counselling.

## Introduction

HIV and infant feeding remains a global public health challenge despite advances in biomedical research. While gains have been achieved in treatment and prevention strategies, prevention of mother-to-child transmission (PMTCT) of HIV continues to be a dynamic and rapidly changing field (1). The 2010 World Health Organization (WHO) infant feeding guidelines reflect significant new evidence and knowledge regarding antiretroviral therapy (ART) and breastfeeding (2). While formula feeding offers the safest postnatal prevention of HIV infection, its implementation in resource-poor settings poses risks of survival among children born from mothers infected with HIV (3). Breastfeeding, especially early initiation and exclusive breastfeeding in the first 6 months, offers protection from postnatal HIV infection (4).

In 2012, the WHO released a revised framework for infant feeding guidelines that were practically the same as the 2010 guidelines regarding breastfeeding, but adding a recommendation that all pregnant mothers should take antiretroviral (ARV) drugs for life (Option B+), coupled with an alternative recommendation for countries to choose Option B, in which a mother could be tested for her eligibility for ARVs after the birth of her child (2). Both the 2010 infant feeding guidelines and the revised framework recommended exclusive breastfeeding for 6 months or beyond, followed by gradual weaning (2). The guidelines continue to highlight the importance of avoiding mixed feeding to reduce the risk of HIV transmission and to avoid diarrhoea and malnutrition, although difficulties have been reported in some resource-poor settings (5) and where some mothers have been reported to prefer exclusive formula feeding regardless of the promotion of exclusive breastfeeding (6). Despite its recognised importance, exclusive breastfeeding is not widely practiced in the developing world (7); although a study conducted among HIV-positive mothers in Tanzania showed greater duration of exclusive breastfeeding, the results fail short of national and international recommendations (8). Factors such as the level of education, knowledge of exclusive breastfeeding in relation to PMTCT, the number of infant feeding counselling sessions attended and initiation of breastfeeding within 1 h of giving birth impact exclusive breastfeeding practice in some settings of the sub-Saharan Africa (9). Researchers have observed that mothers enter the PMTCT programme with pre-existing views on the best way to feed their infants and, therefore, overcoming challenges of adherence to the infant feeding advice is dependent on adequate counselling and clear communication of the rationale for the recommended practices (10). However, the WHO recognises that there are settings where replacement feeding may remain the best strategy to promote HIV-free survival for HIV-exposed infants (2).

In Zambia, mother-to-child transmission (MTCT) of HIV during pregnancy, delivery or breastfeeding is one of the key drivers of the epidemic, in which prevalence of HIV among women in the reproductive age of 15–49 years is 15% (11). Efforts to reduce MTCT have shown improvements in the rate of transmission from 24% in 2009 to 12% in 2012 (12). The Zambia national PMTCT programme uses an opt-out approach which makes HIV testing part of the routine laboratory processes undertaken during all pregnancies (13). In addition, the 2013 Zambian consolidated guidelines were rolled out to provide comprehensive approaches for reducing new HIV infections, PMTCT and provision of lifelong ART regardless of CD4 count for pregnant and breastfeeding women, for HIV-infected sexual partners of pregnant and breastfeeding women and for HIV-infected partners in sero-discordant couples (14). Regardless of their serostatus, all mothers in Zambia are encouraged to exclusively breastfeed in the first 6 months of infant feeding (13, 14). Although these efforts are in line with the international recommendations for prevention of HIV infection among children with a particular focus on treatment and infant feeding (2), emphasis on retention of HIV-positive mothers in the PMTCT programme by preventing loss to follow-up should be supported by population-specific research evidence. Therefore, the overall aim of this study was to explore HIV-positive mothers’ experiences with exclusive breastfeeding during the first 6 months of the infant's life.

## Methods

This health facility- and community-based exploratory qualitative study was part of a larger study entitled ‘HIV and infant feeding: choices and decision-outcomes in the context of PMTCT among HIV-positive mothers in Zambia’. The study was aimed at enhancing safer infant feeding practices during the first 6 months of the infant's life. Individual interviews were conducted with 30 HIV-positive women. The study comprised four contacts with the participants subsequent to giving birth: at 6 days, 6 weeks, 12 weeks and 18 weeks. The mothers were selected from urban settings that were supplied with clean water and sanitation facilities by the local municipalities. Access to the health facilities providing PMTCT services was within walking distance of 30–90 min.

### Sampling

Purposive sampling was used to select HIV-positive mothers who had been recruited in the PMTCT programme from the selected sites.

#### Inclusion criteria

All HIV-positive mothers were eligible to be selected as study participants. However, to obtain a required sample, we recruited mothers aged 15–24, 25–34 and 35+ years while remaining cognizant of the consenting age (18 years).

Selection criteria for the study were as follows: following counselling and initiation on ARV regimen, the mothers should have opted to exclusively breastfeed for the first 6 months, have been counselled on how to feed their infants, have attended health promotion talks as a prerequisite for recruitment in the study and have given birth to babies at full term (9 months) with normal birth weights (2.5–3.5 kg). At the fourth contact (18 weeks after giving birth), each mother and her infant disengaged from the study and continued receiving care and other services at appropriate health facilities.

#### Sample size

A total of 30 HIV-positive mothers met the selection criteria and were recruited to participate in the study. The sample size was arrived when there was no more new information that came to the fore and saturation was thus achieved.

### Data collection procedures

All the interviews were conducted by the principal investigator, who is the first author, and assisted by midwives who were trained in counselling and PMTCT intervention. Data were collected through individual interviews, which was appropriate for gaining an understanding of personal experiences with exclusive breastfeeding practice in the context of PMTCT. Where mothers gave consent, interviews were conducted at their homes or a place convenient to them. Additional contact opportunities occurred when the mothers reported at health facilities for child growth monitoring and immunisation, enabling observation of participants interacting with health care workers. Observations were also used as a complementary method to prepare the next interview and establish rapport by discovering issues to follow-up in the subsequent individual interviews.

### Data collection tool

Data were collected using a semi-structured interview guide that enabled participants to tell their stories in their own way using their primary languages (Cibemba and Cinyanja). The main questions we asked for the study reported here were the following: 1) how can an HIV-positive mother infect her baby with HIV during breastfeeding? (first visit); 2) why did you choose to feed your baby in this way (exclusive breastfeeding)? (first visit); 3) how are you feeding your baby now? (second, third and fourth visits); 4) what can you tell me about the benefits you derived from feeding your baby? (second, third and fourth visits); and 5) what can you tell me about the way you have been feeding your baby from the time you gave birth until now (experience with opportunities and challenges faced)? (fourth visit).

### Data management

Interviews were tape-recorded and transcribed in full, and field notes of reflexive observations generated were recorded in a research diary. All transcripts were checked by the first author for accuracy and quality, cleaned for anonymity and imported into QRS NVivo Version 10 for coding and analysis.

### Data analysis

We began a framework data analysis right from the first interview and during data collection to ensure that we became familiar with the data from the field. This provided us an opportunity to read and re-read the transcripts and identify initial themes/categories, thereby developing a coding matrix where we assigned data to the themes and categories.

To obtain descriptive accounts, we summarised and synthesised the range and diversity of coded data by refining recurrent and significant themes and categories and identifying associations between the themes until the whole set of phenomena of infant feeding experience in the context of PMTCT emerged. Developing associations/patterns within concepts and themes and reflecting on all the data ensured that participant accounts were accurately presented to avoid misinterpretation. Four major themes emerged, which explained and gave meaning to behavioural processes that determined decision-making and experience with exclusive breastfeeding.

To explore the mothers’ experiences with exclusive breastfeeding, we identified relevant codes to provide a thick description of accounts that informed this article. The thick descriptions were achieved through the development of four subthemes that provided a whole phenomenon of experience with exclusive breastfeeding: 1) MTCT of HIV: the mothers’ perspectives; 2) the mothers’ understanding of benefits of exclusive breastfeeding; 3) exclusive breastfeeding: reflections and narratives in relation to MTCT; and 4) challenges experienced during the first 6 months of the infant's life.

## Results

### Participant information

The median age for the participants who met the selection criteria to participate in this study was 28 years. Almost all mothers reported that they were married, had attained only primary education and were predominantly unemployed.

**Table:** 

Characteristic	Categories	Frequency
Age	15–24	10
	25–34	16
	35 >	4
Marital status	Single	2
	Married	27
	Widowed	1
Education	0–7	14
(Grades)	8 and 9	12
	10–12	2
	College education	2
Employment	None	28
	Employed	2
Children	1–3	21
	4–6	9
Total		30

### MTCT of HIV: mothers’ perspectives

In the settings where the participants were recruited from, health care workers conduct health education talks on various topics, including HIV infection, MTCT of HIV, infant feeding and exclusive breastfeeding. To contextualise the experiences with exclusive breastfeeding, we asked participants to explain how a mother infected with HIV could pass the virus to her baby through breastmilk, and the following participants’ responses highlight their understanding of MTCT of HIV:

If I start giving solid food early the baby can have sores in the stomach and when I breastfeed the baby, he can get infected with HIV. [Participant # 30]

When I am breastfeeding, I need to ensure that there are no cracks or sores on my nipples. If there are sores on the nipples, then blood will be coming out when the baby is breastfeeding and the blood is the one which carries the HIV virus. So, if I have sores I can stop breastfeeding the baby because the baby will suck blood. [Participant # 25]

### Mothers’ understanding of the benefits of exclusive breastfeeding to their babies

While the focus of this study was on experiences with exclusive breastfeeding, it was important also to highlight how participants understood the known benefits of both breastfeeding and exclusive breastfeeding for HIV-exposed infants – information which should have been provided to mothers attending group health education and infant feeding counselling. When mothers referred to exclusive breastfeeding, their emphasis was consistently on following instructions given by health care workers rather than on knowledge acquired on how to reduce the risk of MTCT of HIV through breastmilk. One participant explained: ‘exclusive breastfeeding is based on what we were taught at the clinic so that I can take care of the baby's life and mine’ [participant # 2].

Another participant explained: ‘The nurses were teaching us at the clinic that the baby should be breastfed exclusively for six months without giving him any other foods, only breast milk’ [participant # 21].

Furthermore, to understand behaviour change in practicing exclusive breastfeeding, we explored whether mothers knew the benefits to their HIV-exposed infants. The following subthemes highlight the context in which mothers understood the benefits of breastfeeding and in relation to PMTCT.

### Breastfeeding is good for the baby

For some mothers, breastfeeding their babies was associated with better health outcomes. One participant explained that her baby did not have any health problems because she was only breastfeeding: ‘I am only breastfeeding and the baby does not have any health problems, and I do not have any pressure to look for food for the baby’ [participant # 8].

#### Breastmilk contains all the nutrients and water

In another interview, a participant commented that breastmilk provides all the needed nutrients for the baby, including the fact that it has water as one of its components: ‘All the nutrients needed for the baby up to six months are there in the breastmilk, including water’ [participant # 11].

#### The baby breastfeeds on demand

The knowledge that breastmilk is always available on demand was universal among the mothers interviewed in this study: ‘There are many benefits of breastmilk and one is that it is not scarce and at any time you can give the baby and it is not expensive’ [participant # 11].

### Exclusive breastfeeding: reflections and narratives in relation to MTCT

In this study, we described the infant feeding experience in relation to three sub-themes: 1) anxiety about whether the baby was already born with HIV, 2) concern about HIV-free survival for the exposed infants and 3) social support during infant feeding.

### Anxiety about whether the baby was born with HIV

Despite an understanding of the known benefits of breastfeeding and exclusive breastfeeding, some mothers were particularly concerned that their babies may have been born with HIV. Typical responses were the following:

I am just taking chances to breastfeed … there are times when I think … what if the baby was born with HIV … I am just taking chances. [Participant # 6]

The fear of infecting the baby with HIV through breastmilk is there, but again the baby may have contracted HIV through other ways, maybe in the womb, maybe she could have contracted the virus at birth or any other time. [Participant # 18]

We were given the drugs to protect the baby from HIV infection but it can also happen that the baby may have already been born with HIV and then you breastfeed him. Again if he is not found with HIV, he may have it in future. So I start to think that I should just stop breastfeeding and start formula feeding. [Participant # 23]

Sometimes I worry that my baby can have a problem in future because she could have been born with HIV … or can get infected with HIV through breastmilk. But there is nothing I can do because I have already chosen to breastfeed, whatever will happen I will just continue giving my baby the medicine. [Participant # 24]

### Concern about HIV-free survival for the exposed infants

In infant feeding counselling, the health care workers encourage mothers to take their medicine (ARVs) and to continue exclusive breastfeeding for the first 6 months of the infant's life. However, doubts lingered in the minds of some mothers as to whether their infants were protected by the medicine (ARVs) and what a negative test result would mean for their babies:

I usually have thoughts like if they test him and find that he is negative, will I stop breastfeeding or will I introduce him to formula? It is hard. [Participant # 24]

Another mother expressed deep emotion about how she did not want her baby to end up like her:

Yes, like my HIV-positive status, I do not want my baby to be the way I am. So I want to follow the instructions I was told at the clinic so that I see whether they will help me or not. [Participant # 30]

Some mothers were even more concerned when sores appeared in the mouth of the baby. Out of desperation, one mother planned to express and spill the milk because she did not know what to do:

… for instance, she has sores with blood in the mouth so I was thinking of first expressing and spilling some milk before putting the baby to the breast. I was confused and thinking about different ways of protecting my baby like going to consult the doctor. [Participant # 27]

### Social support during the first 6 months of infant feeding

Having someone around at the time of giving birth and for the first few months of infant feeding was described as a source of support and encouragement. Predominantly, mothers reported having someone around for support such as husbands, partners, sisters, mothers and fellow HIV-positive mothers. The following were some of the responses:

I have the support especially from my husband and from my mother's side and they encourage me so much and that is why my heart feels as though I am not HIV-positive. [Participant # 26]

My family has been very supportive and they ask how the baby is doing and whether I am giving her the medication. [Participant # 18]

Previously, I used to get scared but now we meet with different mothers at the clinic when we are getting the drugs and most of them are breastfeeding and so it encourages me to keep breastfeeding my baby. [Participant # 21]

### Problems experienced during the first 6 months of the infant's life

Despite the efforts by mothers to follow the instructions on infant feeding in relation to PMTCT, problems were experienced. Individual circumstances made it complicated to carry out the decision to practice exclusive breastfeeding. There were concerns about the health of the mother and the baby, and about the need to maintain a healthy diet for the mother, as reflected in the following subthemes.

### The well-being of the mother and the baby

The general well-being of mothers and babies was reported as a challenge. A mother complained of signs of malaria with loss of appetite: ‘I have a problem with malaria and it was quite serious and I could not even eat’. While her own health was a concern, the same mother reported that her baby was also sick:

The baby has diarrhoea, is coughing and sneezing. I have so much fear that the virus that I have may also go to my baby, because he is usually sick of diarrhoea, it doesn't finish for a month and a week now. Even in the night he can have diarrhoea five times and it is greenish in colour. [Participant # 29]

Similarly, another mother explained:

The baby had rash and when I went to the hospital the baby was given medicine but I think the rash is developing again … so I stopped giving her the medication (ARVs). [Participant # 27]

### Maternal nutrition: challenges to access food

Generally, mothers in this study were not economically empowered, and they gave expression to the difficulties faced in ensuring that they had nutritious food:

Yes the demand is there because as a mother you need to ensure that the baby is well-fed but I also need to eat well but sometimes when there is no food I just drink water and it affects the flow of milk. [Participant # 11]

Another mother added:

There are times when things are hard. I get dried maize seeds, I roast them and eat and then I drink water. Luckily milk comes out a lot. There is nothing I can do. [Participant # 27]

However, there were also reports of mothers who managed to have three meals in a day:

We have three meals per day, in the morning we have tea with bread and avocado, lunch is nshima (maize meal) with either meat and vegetables, and then snacks in between. Then I eat supper again. [Participant # 17]

## Discussion

The aim of this study was to explore HIV-positive mothers’ experiences with exclusive breastfeeding during the first 6 months of the infant's life. Our findings indicate that despite the known benefits of exclusive breastfeeding to HIV-exposed infants, mothers in selected populations face challenges in achieving safer feeding practices. In this study, individual circumstances relating to the mothers’ own well-being and that of their babies had a bearing on exclusive breastfeeding practice. Poor feeding practices, such as abrupt weaning, could have impacted child health outcomes, and it highlighted gaps in the way mothers were counselled, prepared for practicing exclusive breastfeeding and follow-up care. Studies conducted in similar settings showed that mothers who were counselled during antenatal care and followed up after delivery were more likely to practice exclusive breastfeeding (15). Mothers in this study received once-off infant feeding counselling and group health education during antenatal care, and their difficulties in coping during infant feeding could have been due to poor understanding of information on exclusive breastfeeding and known cultural practices (16). However, we recognised that lack of available trained health care workers could also hinder the uptake of PMTCT services in this and similar settings of resource-poor countries (17–19).

Research has shown that breastfeeding significantly reduces the risk of malnutrition and serious infectious diseases, especially in the first year of the infant's life, while the absence of breastfeeding during the first 2 months of life is associated with a sixfold increase in child mortality in resource-poor countries due to infectious diseases (3, 20). Although mothers in this study understood that breastmilk had essential elements for the healthy growth of their HIV-exposed infants, they lacked the knowledge of how to care for their breasts in the event that sores developed on the nipples. Therefore, they potentially risked depriving infants of breastmilk with its essential elements for healthy growth and protection from infectious diseases. Studies conducted in Malawi and Kenya suggested that breast complications such as mastitis could pose a risk of MTCT of HIV-1 because it is associated with an opening of paracellular pathways with an increase in inflammatory cells and plasma-derived components that could contain HIV-1 and might have serious consequences for exposed infants (21, 22).

Mothers in this study reported about childhood diseases such as diarrhoea and upper respiratory tract infections that could be associated with poor feeding practices and mixed feeding (5). In settings where mixed feeding is a norm (5, 16), the likelihood exists that mothers may mix breastfeeding with other fluids, traditional medicines, or even foods, which could predispose their babies to infections. Hence, they needed to be informed about the importance of seeking health care early enough to avoid further complications. For optimal child health outcomes, babies exposed to HIV infection require constant monitoring and care. Avoidance of mixed feeding has been discussed widely in the WHO infant feeding guidelines (2, 23).

The complexity of the mothers’ concerns was particularly evident when they expressed anxiety that their babies may have been infected with HIV either during pregnancy or at birth. They needed support from health care workers to ensure that the polymerase chain reaction (PCR) tests were done as required and the results communicated immediately so that they could be clear about their individual circumstances and have their anxiety allayed. Where appropriate, arrangements could have been made for further support of mothers whose babies tested positive. In similar settings, and where mothers experienced problems during the first 6 months of infant feeding, researchers recommended support of mothers during the postnatal period to help overcome pitfalls and challenges (24).

In this study, the mothers reported that they were less stressed in the presence of their social support networks, such as husbands, sisters, mothers and friends – structures that have been known to provide opportunities for improved uptake of PMTCT services in similar settings (25–27). The social support network in this case could have helped to ensure that the mothers’ nutritional needs were taken care of, since the mother's well-being is important for successful infant feeding experience. Researchers recognise healthy mothers to be key to the well-being of their children (9, 28), prompting recommendations to involve family members in infant feeding counselling and education, especially with regard to exclusive breastfeeding practice (9). Social support is possible in societies where extended families are closely knit, and it can easily be integrated into PMTCT, within the national guidelines, to improve the uptake of exclusive breastfeeding.

## Conclusions

We conclude that HIV-positive women face challenges to practice exclusive breastfeeding in selected low-resource settings. Individualised, unbiased and accurate information on exclusive breastfeeding will enhance the safety of infant feeding practices in this and similar settings. To achieve this, a model for follow-up of HIV-positive mothers should be designed to provide health care workers with steps for consideration during counselling.

### Limitations

Although we cannot generalise these findings to a larger cohort, the study nevertheless provides evidence that can inform PMTCT interventions and improve exclusive breastfeeding practices and follow-up of HIV-positive mothers. The interviews of this study were conducted in the primary languages of the participants, and all data were translated into English for analysis; we acknowledge that nuances of meaning may have been lost during the translation process despite the fact that the first author verified the translation with the research assistants.

## Paper context

Breastfeeding, especially early initiation and exclusive breastfeeding in first 6 months, offers protection from postnatal HIV infection for exposed infants. Mothers infected with HIV in this and similar settings face challenges to practice exclusive breastfeeding. Individualised, unbiased and accurate information will enhance the safety of exclusive breastfeeding practices in selected populations. Therefore, a model for follow-up of HIV-positive mothers should be designed to provide frontline health care workers with steps for consideration during infant feeding counselling.
